# Adipocytes promoted anoikis resistance of head and neck cancer cells via overexpressed ITGA5

**DOI:** 10.1186/s40001-025-03252-5

**Published:** 2025-10-17

**Authors:** Yurong He, Yueyue Shi, Xinyu Li, Ru Wang, Lingwa Wang, Haiyang Li, Siyu Zhu, Shizhi He, Jugao Fang

**Affiliations:** 1 Department of Otorhinolaryngology Head and Neck Surgery, Beijing Tongren Hospital, Capital Medical University, No.1, Dongjiaominxiang Street, Dongcheng District, Beijing, 100730 People’s Republic of China; 2 Ministry of Education, Key Laboratory of Otorhinolaryngology Head and Neck Surgery (Capital Medical University), No.1, Dongjiaominxiang Street, Dongcheng District, Beijing, 100730 People’s Republic of China

**Keywords:** Head and neck squamous cell carcinoma, Integrin subunit α5, Anoikis, Adipocytes

## Abstract

**Background:**

Head and neck squamous cell carcinoma (HNSCC) was the sixth most common cancer worldwide. Given the evidence that adipocytes are related to the progression of various cancers, this study explored the internal connection and mechanism between HNSCC and adipocytes.

**Methods:**

Quantitative real-time PCR (qRT-PCR) was used to examine the transfection efficiency of integrin subunit α5 (ITGA5) overexpression plasmid and small interfering RNA of ITGA5 (si-ITGA5). Colony formation assay was employed to detect the colony formation rate of FaDu and SAS cells, while flow cytometry was used to measure cell apoptosis rate. The protein levels of cleaved-caspase-3 (apoptosis marker) and cell adhesion markers (ITGA5, integrin subunit β1 (ITGB1) and cluster of differentiation 44 (CD44)) were analyzed by western blotting. After HNSCC cells and adipocytes were co-cultured, the last three experiments above-mentioned were repeated and Calcein AM/EthD-1 double stain assay was performed to examine anoikis.

**Results:**

Overexpressed ITGA5 enhanced colony formation and inhibited anoikis in HNSCC cells (*p* < 0 *p* < .001), while reducing the expression of the apoptosis-related protein and increasing those of adherence-related proteins (*p* < 0.05). However, si-ITGA5 had the opposite effect. After co-culture of HNSCC cells with adipocytes, cell colony formation was increased, anoikis was inhibited and the expressions of ITGA5 and cell adhesion markers were upregulated (*p* < 0.05). The effects of HNSCC cells co-cultured with adipocytes on colony formation, anoikis and cell adhesion markers were reversed by ITGA5 silencing (*p* < 0.01).

**Conclusion:**

Adipocytes promote anoikis resistance of head and neck cancer cells via overexpressed ITGA5.

## Introduction

Head and neck squamous cell carcinoma (HNSCC) is the sixth most common cancer worldwide, with up to 600,000 new cases annually [1]. Complex and changeable social environment and lifestyle may lead to high body mass index (BMI), heavy smoking, human papilloma virus (HPV) infection and excessive alcohol consumption, thus increasing the probability of HNSCC [2–5]. In recent years, effective treatment methods for HNSCC are mainly attributed to the continuous improvement of medical technology, with radiotherapy, surgery and pharmacotherapy remaining the cornerstone interventions [6]. However, studies indicated that HNSCC patients have a poor prognosis, with nearly 50% experiencing disease recurrence after primary treatment. The high rate of treatment failure is primarily attributed to metastasis and local recurrence [7–10]. Consequently, there is an urgent need to develop new and effective methods to improve the survival outcomes of HNSCC patients.

Anoikis is a type of internal apoptosis triggered by loss of adhesion or improper adhesion to the extracellular matrix (ECM) [11]. Resistance to anoikis is a prerequisite for tumor metastasis, and ample studies have confirmed that regulating anoikis is of great significance to promote cell metastasis in a variety of cancers, including but not limited to gastric cancer, lung cancer, colorectal cancer and HNSCC [12–15]. Previous studies have revealed that cancer cells may develop anoikis resistance to facilitate metastasis [15]. Of note, epidermal growth factor (EGF) promotes anoikis resistance and tumor metastasis in HNSCC by inducing the expression of angiopoietin-like 4 (ANGPTL4) [15]. In addition, multiple mechanisms, including the involvement of adhesion molecules, death receptors, and integrin receptors, can drive anoikis resistance and the development of metastatic cancer cells [16].

Integrin subunit α5 (ITGA5) is a heterodimer of α5β1, binding to integrin subunit β1 (ITGB1) [17]. Integrins serve as a class of heterodimer integral membrane proteins that mediate not only cell adhesion but also signal transduction [18]. The integrin signaling pathway presents abnormally high activity, demonstrating its necessity for the prevention of anoikis in tumor cells [19, 20]. Notably, ITGA5 has been reported to facilitate anoikis in hepatocellular carcinoma (HCC) and inhibit anoikis in rat bone marrow mesenchymal stem cells (rBMSCs) [21, 22]. However, there are still few studies on the effect of ITGA5 on HNSCC, so we investigated the underpinning mechanism.

## Methods

### Cell culture

Human hypopharyngeal squamous cell carcinoma FaDu cells (HTB-43) were purchased from American Type Culture Collection (ATCC, USA), and human tongue squamous cell carcinoma cells SAS (CL-0849) were ordered from Procell system (China). Human omental tissue-derived adipocytes (KCB2010101) were procured from Kmcellbank (China). FaDu cells were cultured in Eagle's Minimum Essential Medium (EMEM, 30-2003) supplemented with 10% fetal bovine serum (FBS, 30-2020). SAS cells and adipocytes were incubated in Dulbecco's Modified Eagle's Medium (DMEM, 30-2020) incorporating 10% FBS. All culture media and reagents were obtained from ATCC (USA). In addition, FaDu and SAS cells were incubated at 37 °C in a humidified 5% CO_2_ incubator (E2398, Beyotime, Shanghai, China). All cells were analyzed for short tandem repeats and mycoplasma detection.

### Cell grouping

After deliberation, the study was divided into three parts. In the first part, the cells were grouped as follows: control group, normally cultured FaDu and SAS cells; vector group, cells transfected with empty vector; ITGA5 group, cells transfected with ITGA5 overexpression plasmid; negative control of the small interfering RNA (si-NC) group, cells transfected with si-NC; small interfering RNA of ITGA5 (si-ITGA5) group, cells transfected with si-ITGA5. In the second part, cell grouping was as follows: adipocyte group, normally cultured adipocytes; FaDu + adipocyte group, adipocytes co-cultured with FaDu cells; and SAS + adipocyte group, adipocytes co-cultured with SAS cells. In the third part, the cells were grouped as follows: FaDu + adipocyte group, adipocytes co-cultured with FaDu cells; FaDu + adipocyte + si-NC group, adipocytes co-cultured with si-NC-transfected FaDu cells; FaDu + adipocyte + si-ITGA5, adipocytes co-cultured with si-ITGA5-transfected FaDu cells; SAS + adipocyte group, adipocytes co-cultured with SAS cells; SAS + adipocyte + si-NC group, adipocytes co-cultured with si-NC-transfected SAS cells; SAS + adipocyte + si-ITGA5, adipocytes co-cultured with si-ITGA5-transfected SAS cells.

For co-cultured experiment, matrigel (Corning, New York, USA) was diluted with cell culture medium to a concentration of 200 μg/mL and then added to a 24-well plate. FaDu or SAS cells (4 × 10^4^ cells/well) were seeded in an anchorage-resistant culture plate, the transwell chamber was inserted, and adipocytes (4 × 10^4^ cells/well) were planted on the upper side of the chamber. FaDu or SAS cells were co-cultivated with adipocytes through transwell systems for 24 h.

### Quantitative real-time PCR (qRT-PCR)

Total RNA was extracted from FaDu and SAS cells by Trizol Reagent (15,596,018, Romics, Shanghai, China), and transcribed into cDNA by PrimeScript RT Reagent Kit (Perfect Real Time) (RR037B, Takara Biotechnology, Japan). SYBR Green Realtime PCR Master Mix (XY-9A-QPK-201, XYBiotechnology, Shanghai, China) was adopted with the ABI 7500 thermocycler (Shanghai Tusen Vision Technology, China) for detecting the expression of ITGA5. The primers (5′ to 3′) are listed as follows: ITGA5 (F) AGCCTCAGAAGGAGGAGGAC, (R) TTAATGGGGTGATTGGTGGT; beta Actin (β-actin) (F) GCTCTTTTCCAGCCTTCCTT, (R) GAGCCAGAGCAGTGATCTCC. As the reference gene, β-actin was used to normalize the relative expression of ITGA5. The experiment was repeated in triplicate and the relative fold-change was calculated by the 2^−ΔΔCt^ method [23].

### Cell transfection

Prior to cell transfection, si-ITGA5 (C01004, target sequence: TGGTTTCACAGTGGAACTTCAGC) and si-NC (A06001) were obtained from GenePharma (Shanghai, China). Besides, ITGA5 overexpression plasmid was constructed using pcDNA vector (12,489,019, Thermo Fisher, USA) and the empty vector was regarded as the negative control. The above plasmids and oligonucleotides were transfected into FaDu and SAS cells using Lipofectamine^™^ 3000 reagent (13,778,030, Thermo Fisher, Waltham, USA), when cells were seeded to approximately 80% confluence in a 96-well plate. Opti-MEM^™^ medium (31,985,062, Thermo Fisher, USA) was used to dilute Lipofectamine^™^ 3000 reagent and mixed well. Subsequently, diluted siRNA, ITGA5 overexpression plasmid vectors, and empty vector were individually mixed with diluted Lipofectamine^™^ 3000 reagent and later incubated at room temperature for 10 min. Next, all the lipid complexes were separately added to cells for 48-h culture at 37 °C. Finally, the transfection efficiency was analyzed by qRT-PCR.

### Oil red staining assay

The adipocytes were detected by Oil Red O Stain Kit (G1262, Solarbio, Beijing, China) consisting of oil red O fixative, oil red O staining solution, mayer hematoxylin staining and oil red O buffer. The cell culture medium was removed, and the cells obtained were washed twice with PBS and fixed with oil red O fixative for 25 min. Then, the cells were immersed in the newly prepared oil red O staining solution for 20 min, before which the used liquid was removed away. After washing 4 times until there was no more staining solution, mayer hematoxylin staining solution was added for culture of 1–2 min. Cells were washed 4 times and cultivated with oil red O buffer for 1 min. The buffer was discarded, and the cells were covered with distilled water and examined under the microscope (400 × or 200 × , DMi8, Leica, Germany).

### Colony formation assay

First of all, the processed and cultured FaDu and SAS cells were collected. Next, the cells were resuspended in culture medium and seeded into 6-well plates at a density of 1000 cells per well, followed by incubation at 37 °C with 5% CO_2_. Two weeks later, the cells were fixed with 4% paraformaldehyde and subsequently stained with 0.2% crystal violet for 30 min and the colony numbers were observed under a microscope (DMi8, Leica, Germany) to calculate the colony formation rate.

### Western blotting analysis

Cellular proteins were obtained with WB Super RIPA Lysis Buffer (XY9501, XY Biotechnology, Shanghai, China). Subsequently, the concentration of protein in the samples including ITGA5, ITGB1, cluster of differentiation 44 (CD44), cleaved-caspase-3 and β-actin were reliably measured by BCA Assay Kit (P0012, Beyotime, Shanghai, China) based on the manufacturer’s protocol. Equal concentrations of apoptosis-related protein samples Cleaved-caspase-3 and adherence-related proteins including ITGA5, ITGB1, CD44 and the housekeeping gene β-actin were electrophoresed in 6% sodium dodecyl sulfate–polyacrylamide (SDS-PAGE) gels (P0676-500 ml, Beyotime, China), which were transferred onto PVDF membranes (10,600,023, Cytiva, Shanghai, China). The membranes were incubated with primary antibodies anti-Cleaved Caspase-3 (ab32042, 1:500, Rabbit, 17 kDa, Abcam, UK), anti-Integrin alpha 5 (ab150361, 1:4000, Rabbit, 150 kDa, Abcam, UK), anti-Integrin beta 1 (ab52971, 1:5000, Rabbit, 88 kDa, Abcam, UK), anti-CD44 (ab189524, 1:1000, 82 kDa, Rabbit, Abcam, UK) and anti-β-actin (housekeeping gene; ab6276, 1:9000, Mouse, 42kDA, Abcam, UK) at 4 °C overnight. After washing with TBST (B1009-TBST, APPLYGEN, Beijing, China) 3 times, the membranes were incubated with their corresponding secondary antibody Goat Anti-Rabbit IgG H&L (ab205718, 1:3000, Abcam, UK) and Goat Anti-Mouse IgG H&L (ab205719, 1:7000, Abcam, UK) at room temperature for 1 h. The intensity of protein band was visualized using an Efficient Chemiluminescence (ECL) Kit (10,145-100 ml, Proand, Xi'an, China) by ImageJ software with an ImageQuant densitometric scanner (BH-2, OLYMPUS, Japan). Every single experiment was repeated three times.

### Anoikis assay

Live & Dead^™^ Viability/Cytotoxicity Assay Kit for Animal Cells (Calcein AM, EthD-1) (L6023M, UElandy, Suzhou, China) was used to detect Anokis. First, the reagents were naturally maintained to room temperature. Then, 20 μL 2 mM EthD-I and 5 μL 4 mM Calcein AM were stirred with 10 mL phosphate buffered solution (PBS, PBS-C520, Biocomma, Shenzhen, China) to mix evenly. Afterwards, the cells were thoroughly cleaned with 1 × PBS thrice and suspended with 0.5 mL of staining solution. The cell density was controlled to 3 × 10^5^/mL. Then cells were incubated at 37ºC protected from the light for 15 min. Within 2 h, the cell activity was examined with a fluorescent microscopy (200 × , CytoFLEX, BECKMAN COULTER, USA) to measure green Calcein AM fluorescence with emission wavelength at 517 nm and excitation wavelength at 494 nm or red EthD-1 fluorescence with emission wavelength at 617 nm and excitation wavelength at 528 nm.

### Cell apoptosis assay

The apoptosis of transfected cells was examined with Annexin V-FITC Apoptosis Staining/Detection kit (ab14085, Abcam, UK). In brief, 4 × 10^5^ cells were collected by centrifugation and re-suspended with 500 μL 1 × binding buffer. 5 μL Annexin V-FITC and the same dose of propidium iodide (PI) were added (darkness, 5 min, room temperature) for flow cytometry assay (BriCyte E6, Mindray, Shenzhen, China). Adherent cells were washed with serum-containing medium and gently trypsinized for detachment.

### Statistical analysis

The statistical analyses were performed using Graphpad 8.0 software. All experimental data were expressed as mean ± standard deviation. Independent-samples test analysis was conducted for analyzing differences between two groups. One-way ANOVA was conducted for analyzing differences among multiple groups. *P-values* that was less than 0.05 were deemed as statistically significant.

## Results

### Overexpressed ITGA5 accelerated cell colony formation and inhibited apoptosis in FaDu and SAS cells

**Fig. 1:**
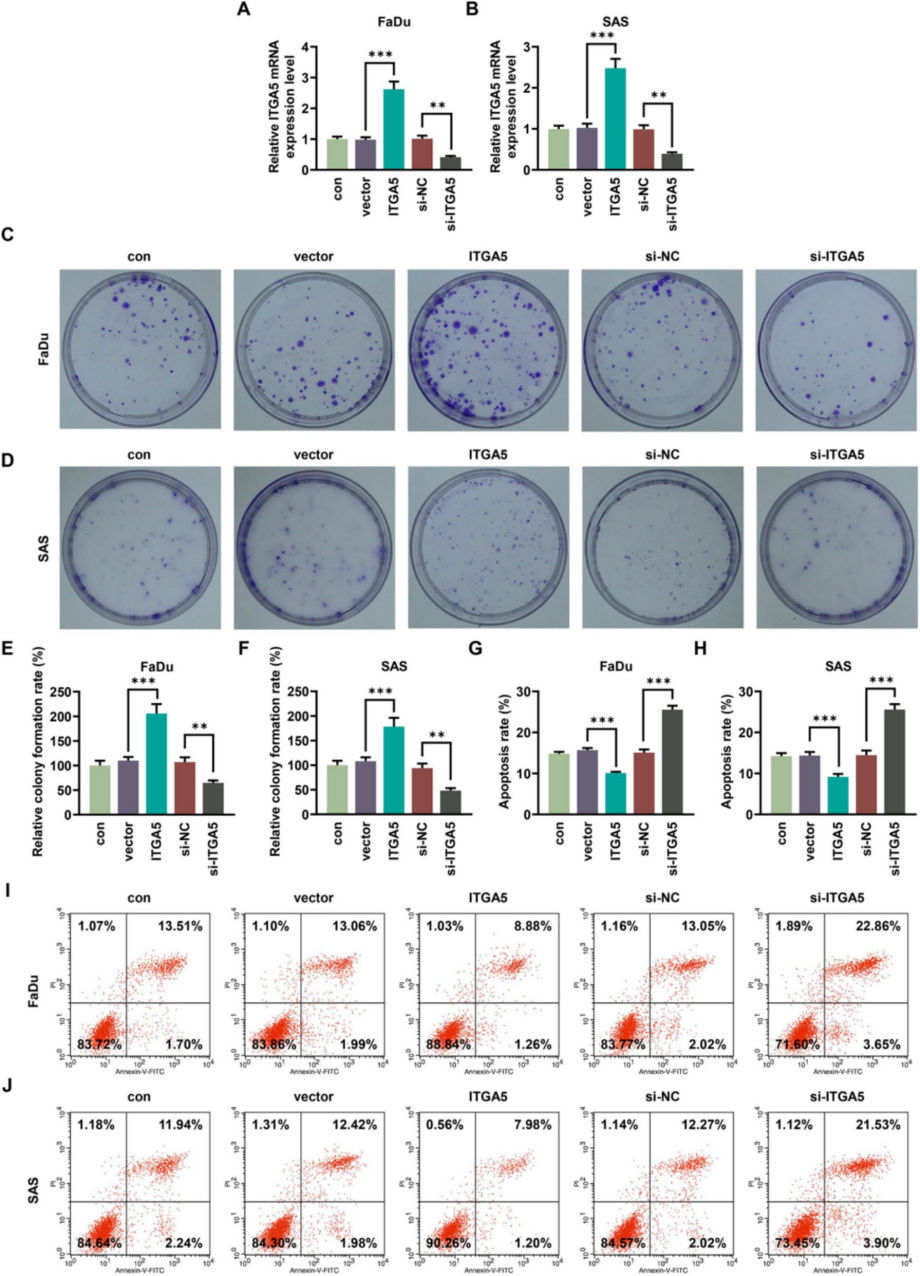
The impact of overexpressed ITGA5 on cell colony formation and apoptosis in HNSCC cells. **A**, **B** After the transfection of si-ITGA5 and ITGA5 overexpression plasmid, the expression of ITGA5 in HNSCC cells including FaDu and SAS cells was determined by qRT-PCR, with β-actin serving as the internal reference. **C–F** The relative colony formation rate was explored by colony formation assay. **G**–**J** Flow cytometry was carried out to measure the apoptosis rate. The upper right (UR) quadrant: the late apoptosis. The lower right (LR) quadrant: the early apoptosis. All experiments were repeated three times to obtain average values. The data are presented as the mean ± standard deviation of three independent experiments; ^**^p < 0.01; ^***^p < 0.001. *HNSCC* head and neck squamous cell carcinoma, *si-ITGA5* small interfering RNA of ITGA5, *si-NC* negative control of the small interfering RNA, *QRT-PCR* quantitative real-time PCR

Firstly, empty vector, ITGA5 overexpression plasmid (ITGA5), si-NC and si-ITGA5 were separately transfected into FaDu and SAS cells. The relative ITGA5 mRNA expression in ITGA5 group was approximately 2.5-fold higher than that in the vector group, and reduced threefold compared to that in the si-NC group, indicating successful transfection (*p* < 0.01, Fig. 1A, B). The colony formation rate in the ITGA5 overexpression group was approximately two-fold higher than that in the vector group (*p* < 0.001), whereas the rate in the si-ITGA5 group was approximately two-fold lower than that in the si-NC group (*p* < 0.01, Fig. 1C–F). According to data of flow cytometry, the apoptosis rate was elevated approximately 1.6-fold in the si-ITGA5 group versus the si-NC group; conversely, it was reduced approximately 1.5-fold in the ITGA5 group versus the vector group (*p* < 0.001, Fig. 1G–J).

### Overexpressed ITGA5 reduced the expression of the apoptosis-related protein and increased the expressions of cell adhesion markers

**Fig. 2:**
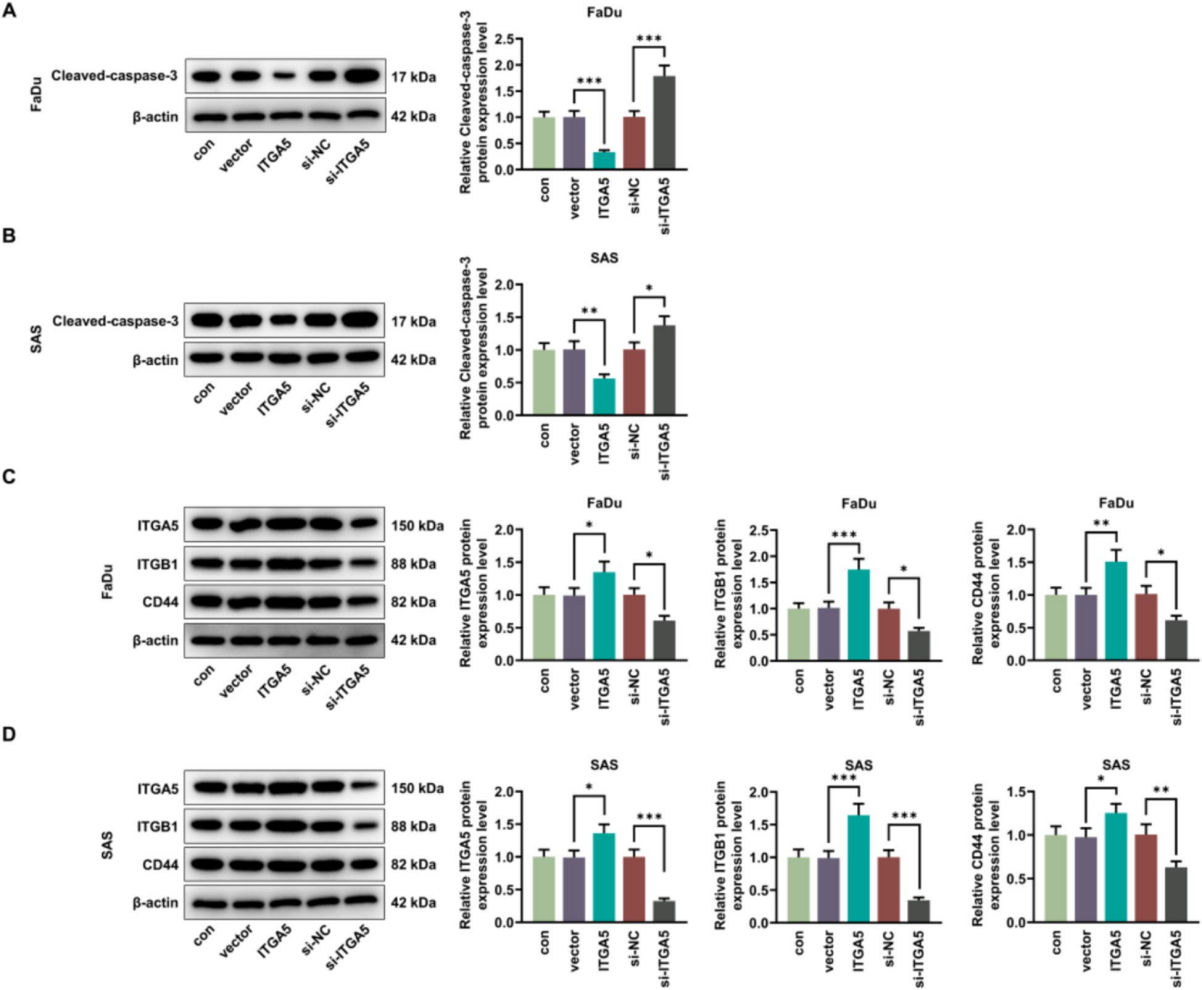
The impacts of overexpressed ITGA5 on apoptosis-related protein and cell adhesion markers in HNSCC cells. **A**, **B** The apoptosis-related protein cleaved-caspase-3 was detected by western blotting analysis after ITGA5 overexpression plasmid and si-ITGA5 transfection, with β-actin serving as the housekeeping gene. **C**, **D** The cell adhesion markers including ITGA5, ITGB1 as well as CD44 were detected by western blotting analysis after ITGA5 overexpression plasmid and si-ITGA5 transfection, with β-actin serving as the housekeeping gene. All experiments were repeated three times to obtain average values. The data are presented as the mean ± standard deviation of three independent experiments; ^*^p < 0.05; ^**^p < 0.01; ^***^p < 0.001. *HNSCC* head and neck squamous cell carcinoma, *ITGA5* Integrin subunit α5, *ITGB1* integrin subunit β1, *CD44* cluster of differentiation 44, *si-ITGA5* small interfering RNA of ITGA5, *si-NC* negative control of the small interfering RNA, *QRT-PCR* quantitative real-time PCR

In the FaDu and SAS cells, the apoptosis-related protein (cleaved-caspase-3) expression in ITGA5 group was approximately 2–threefold lower than that in the vector group (*p* < 0.01, Fig. 2A, B). However, the expression in si-ITGA5 group was approximately 1.5 fold higher than that in the si-NC group (*p* < 0.05, Fig. 2A, B). In terms of cell adhesion markers including ITGA5, ITGB1 as well as CD44, their expression levels showed an approximately 1.2–1.8 fold increase in the ITGA5 group compared to the vector control, but an approximately twofold decrease in the si-ITGA5 group compared to the si-NC group (*p* < 0.05, Fig. 2C, D).

### After co-culture of HNSCC cells with adipocytes, cell colony formation was increased, anoikis was inhibited and the expressions of ITGA5 and cell adhesion markers were upregulated

**Fig. 3:**
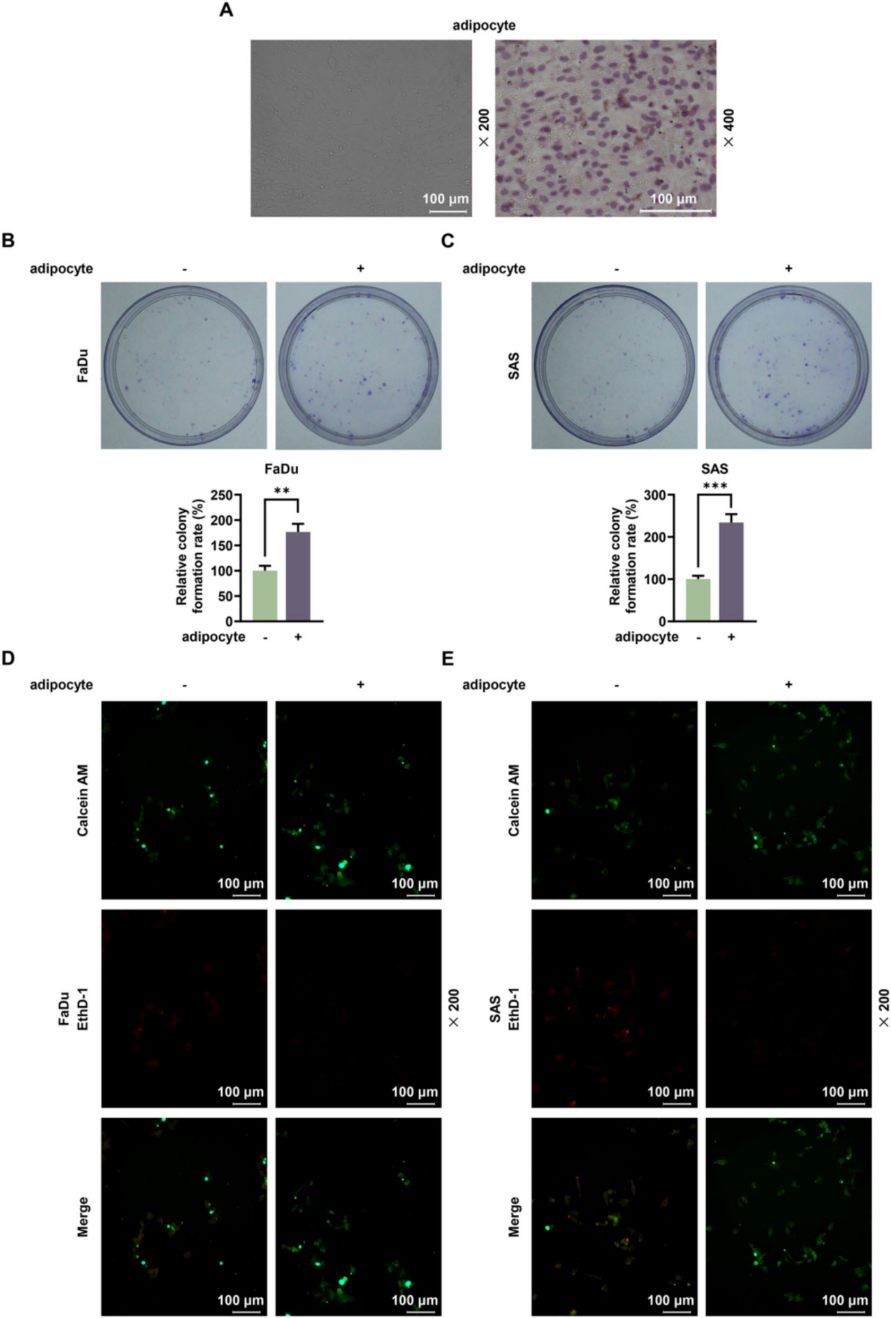
The impacts of HNSCC cells co-cultured with adipocytes on cell colony formation and anoikis. FaDu and SAS cells were co-cultured with adipocytes, and cells without co-culture of adipocytes acted as the control. **A** Adipocytes were investigated by Oil Red O Stain Kit. Magnification: 200 × or 400 × , scale: 100 μm. **B**, **C** The relative colony formation rate was measured by colony formation assay after the co-culture of HNSCC cells and adipocytes. **D**, **E** Calcein AM/EthD-1 staining was performed to detect anoikis after the co-culture of HNSCC cells and adipocytes. Magnification: 200 × , scale: 100 μm. All experiments were repeated three times to obtain average values. The data are presented as the mean ± standard deviation of three independent experiments;^**^p < 0.01; ^***^p < 0.001

**Fig. 4:**
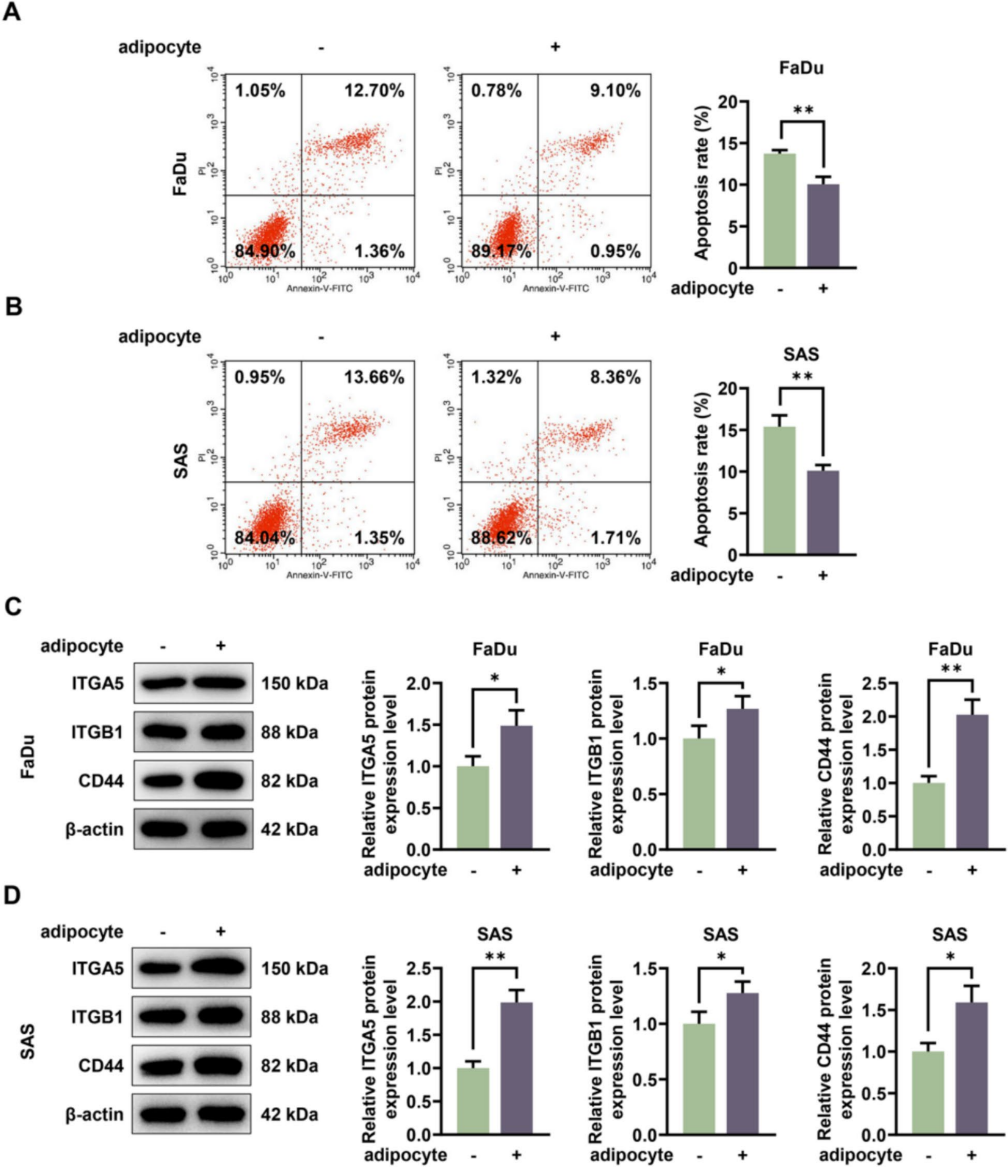
The impacts of HNSCC cells co-cultured with adipocytes on anoikis and cell adhesion markers. FaDu and SAS cells were co-cultured with adipocytes, and cells not co-cultured with adipocytes acted as the control. **A**, **B** Flow cytometry was carried out to measure the apoptosis rate. The upper right (UR) quadrant: the late apoptosis. The lower right (LR) quadrant: the early apoptosis. All experiments were repeated three times to obtain average values. **C**, **D** The adherence-related proteins including ITGA5, ITGB1 as well as CD44 were detected by western blotting analysis after ITGA5 overexpression plasmid transfection, with β-actin serving as the housekeeping gene. All experiments were repeated three times to obtain average values. The data are presented as the mean ± standard deviation of three independent experiments; ^*^p < 0.05; ^**^p < 0.01. *HNSCC* head and neck squamous cell carcinoma, *ITGA5* Integrin subunit α5, *ITGB1* integrin subunit β1, *CD44* cluster of differentiation 44, *QRT-PCR* quantitative real-time PCR

In addition, adipocytes were identified by Oil Red O Stain Kit under the microscope (Fig. 3A). The experimental results showed that co-culture with adipocytes resulted in an approximately twofold increase in the colony formation of HNSCC cells compared to monoculture (*p* < 0.01, Fig. 3B, C). Calcein AM/EthD-1 staining was performed to determine anoikis after co-culture, and the image was presented by fluorescent microscope clearly, suggesting that the anoikis was decreased after HNSCC cells were co-cultured with adipocytes (Fig. 3D, E). The results of flow cytometry revealed that the apoptosis rate was decreased 1.5 fold after HNSCC cells were co-cultured with adipocytes (*p* < 0.01, Fig. 4A, B). Co-culture with adipocytes significantly upregulated the expression levels of ITGA5, ITGB1 and CD44 in both FaDu and SAS cell lines (*p* < 0.05, Fig. 4C, D). The increase ranged from approximately 1.2-fold to twofold compared to their respective monoculture controls.

### The effects of HNSCC cells co-cultured with adipocytes on cell colony formation, anoikis and the expressions of ITGA5 and cell adhesion markers were reversed by ITGA5 silencing

**Fig. 5:**
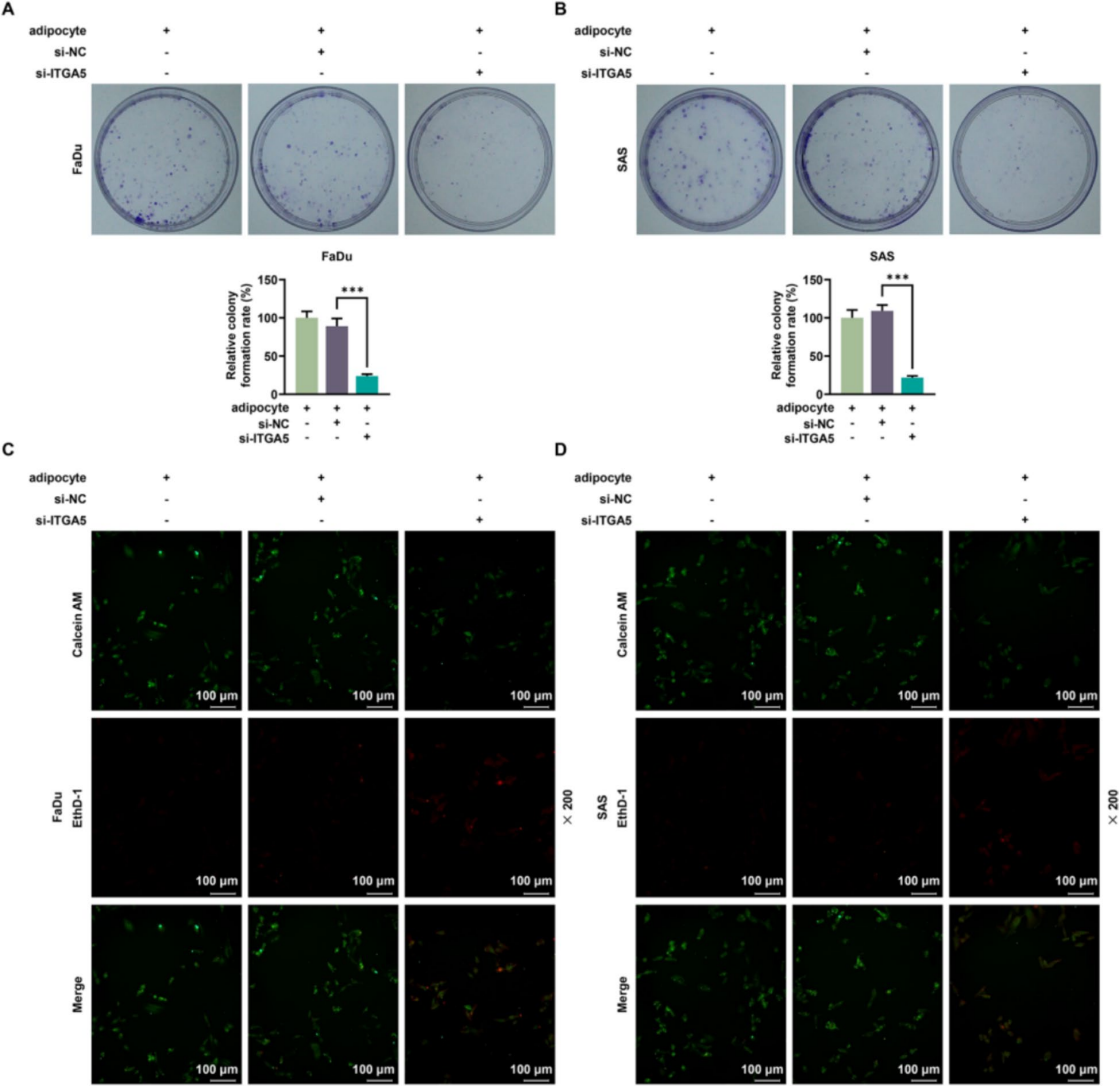
The impacts of HNSCC cells co-cultured with adipocytes on cell colony formation were reversed by ITGA5 silencing. FaDu and SAS cells were co-cultured with adipocytes, and cells were transfected with si-NC or si-ITGA5. **A**, **B** The relative colony formation rate was measured by colony formation assay after the co-culture of HNSCC cells and adipocytes. **C**, **D** Calcein AM/EthD-1 staining was performed to detect anoikis after co-culture. All experiments were repeated three times to obtain average values. The data are presented as the mean ± standard deviation of three independent experiments; ^***^p < 0.001. *HNSCC* head and neck squamous cell carcinoma, *si-ITGA5* small interfering RNA of ITGA5, *si-NC* negative control of the small interfering RNA

**Fig. 6:**
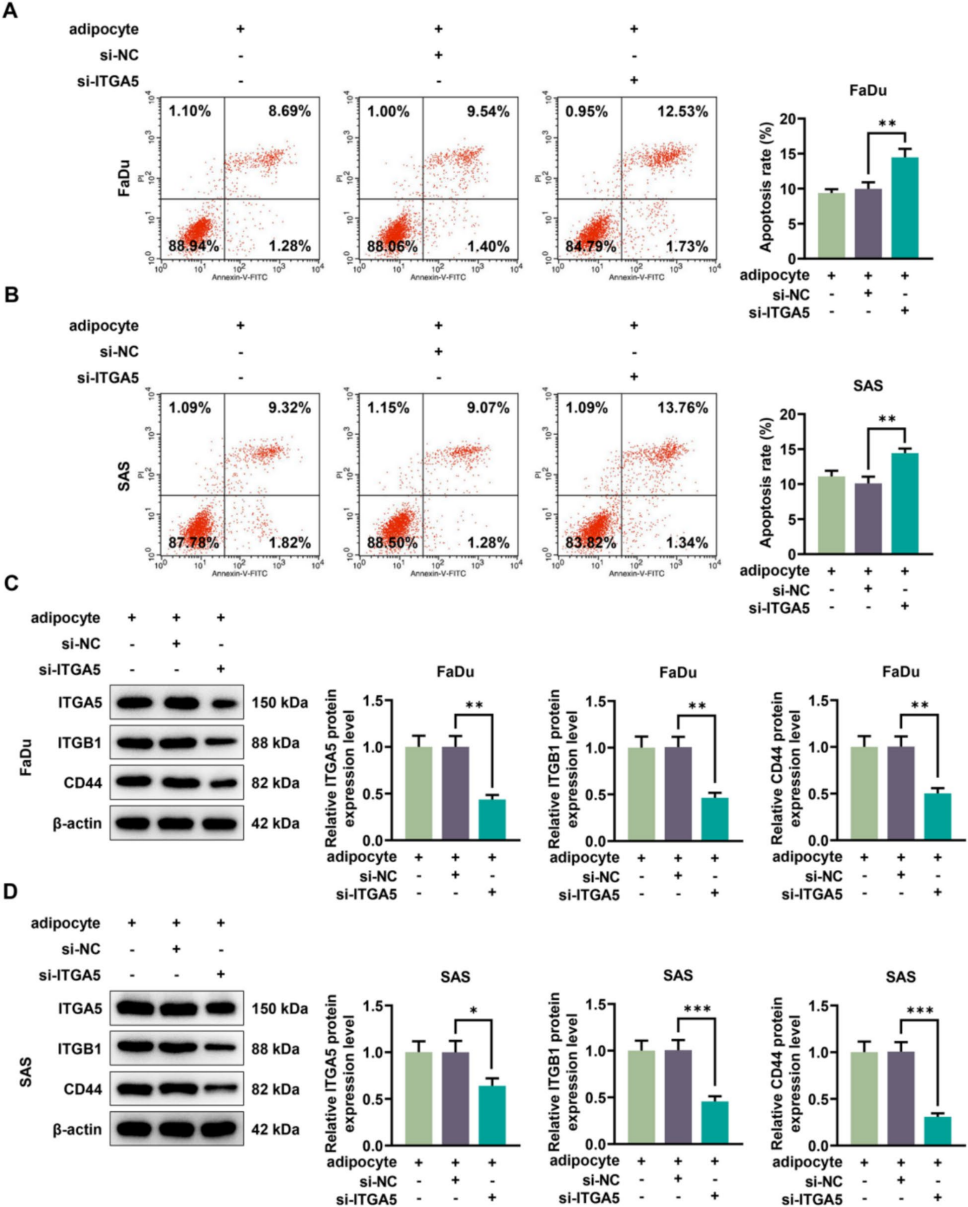
The impacts of HNSCC cells co-cultured with adipocytes on anoikis and cell adhesion markers were offset by ITGA5 silencing. FaDu and SAS cells were co-cultured with adipocytes, and the cells were transfected with si-NC or si-ITGA5. **A**, **B** Flow cytometry was conducted to measure the apoptosis rate. The upper right (UR) quadrant: the late apoptosis. The lower right (LR) quadrant: the early apoptosis. **C**, **D** The cell adhesion markers including ITGA5, ITGB1 as well as CD44 were detected by western blotting analysis after si-ITGA5 transfection, with β-actin serving as the housekeeping gene. All experiments were repeated three times to obtain average values. The data are presented as the mean ± standard deviation of three independent experiments; ^*^p < 0.05; ^**^p < 0.01; ^***^p < 0.001. *HNSCC* head and neck squamous cell carcinoma, *ITGA5* Integrin subunit α5, *ITGB1* integrin subunit β1, *CD44* cluster of differentiation 44, *si-ITGA5* small interfering RNA of ITGA5, *si-NC* negative control of the small interfering RNA, *QRT-PCR* quantitative real-time PCR

The relative colony formation rate was measured by microscope with colony formation assay, and the results indicated that the relative colony formation rate of adipocyte + si-ITGA5 group was approximately decreased fivefold compared with adipocyte + si-NC group (*p* < 0.001, Fig. 5A, B). Calcein AM/EthD-1 staining was performed to detect anoikis after co-culture, and the image was presented by fluorescent microscope, which implied that si-ITGA5 reversed the reduction of anoikis after co-culture of HNSCC cells with adipocytes. (Fig. 5C, D). Furthermore, the results of flow cytometry demonstrated that the apoptosis rate in adipocyte + si-ITGA5 group was approximately increased 1.5 fold compared with adipocyte + si-NC group, suggesting si-ITGA5 abrogated the decline of apoptosis rate due to co-culture of HNSCC cells with adipocytes (*p* < 0.01, Fig. 6A, B).

In light of western blotting data, the expressions of ITGA5, ITGB1 and CD44 in adipocyte + si-ITGA5 group were approximately reduced twofold relative to adipocyte + si-NC group in FaDu cells, whilst their expressions in adipocyte + si-ITGA5 group were approximately reduced 2.5 fold in contrast with adipocyte + si-NC group in SAS cells, denoting that si-ITGA5 offset the impact of adipocytes co-cultured with HNSCC cells (*p* < 0.05, Fig. 6C, D).

## Discussion

HNSCC is a commonly diagnosed and highly aggressive cancer globally, representing one of the most deadly malignancies [24]. Despite current medical advances and research results, managing cancer-related pain and improving quality of life remain major challenges in the care of patients with HNSCC.

ITGA5 is intimately associated with poor overall survival and relapse-free survival in HNSCC [25]. As we all know, ITGA5 is of vital significance in cancer research, which belongs to the integrin family. Integrin, a cell adhesion receptor, consists of about 24 α and β subunits [26]. ITGA5 plays a central role in proliferation, invasion and metastasis, most typically in multiple types of cancers [27, 28], including HNSCC [29]. Additionally, several studies have demonstrated that ITGA5 modulates anoikis to influence the proliferation and migration of varying cancers [21, 22]. For example, loss/gain-of-function assays revealed that ITGA5 plays a critical role in anoikis in HCC cells [21]. Separately, another study found that ITGA5 also hampers anoikis and accelerates the proliferation and migration of rat bone marrow mesenchymal stem cells (rBMSCs) [22]. Therefore, we assumed that ITGA5 also regulated anoikis to promote HNSCC development. To clarify the role of ITGA5 in HNSCC, we selected FaDu and SAS cells for cell modeling. According to the specific experimental data, overexpressed ITGA5 could promote colony formation and inhibit anoikis, whereas silenced ITGA5 exerted opposite effects.

Anoikis is a specific pattern of apoptosis triggered by inadequate cellular matrix interactions [30]. In fact, cancer cells would adopt a variety of strategies to suppress the metastasis caused by anoikis, which is called anoikis resistance [31]. Beyond established methods including the involvement of adhesion molecules and integrin receptors such as ITGA5, new approaches are still being explored [16]. Recent research confirmed that obesity suppresses the sensitivity of gastric cancer to anoikis and enhances metastasis [32]. Besides, adipocytes co-incubated with gastric cancer cells have the unexpected effect of preventing the cells from anoikis and expediting gastric cancer omental metastasis [33]. Therefore, we preliminarily speculated that co-culture of adipocytes and HNSCC might also impact anoikis.

Adipocytes are derived from bone marrow mesenchymal stem cells (BMMSCs), which present in bone marrow, adipose tissue as well as dental pulp [34, 35]. Some studies have confirmed that obesity is closely related to the generation and aggravation of various cancers; for instance, obesity enhances oral cancer progression [36]. Moreover, adipose tissues promote metastasis of HNSCC [37]. As an indispensable marker of adherence-related protein, CD44 could enhance metastatic potential in malignancy [38]. ITGA5 and ITGB1 could influence anoikis, and were also implicated in the experiment of exploring anoikis [22]. In recent years, elevated cleaved caspase-3 expression has been found to promote apoptosis [39]. In our study, HNSCC cells co-cultured with adipocytes boosted cell colony formation, inhibited anoikis, and increased the expressions of cell adhesion markers including ITGA5, ITGB1 and CD44, which were reversed by ITGA5 silencing. Briefly, adipocytes promoted anoikis resistance of HNSCC via overexpressed ITGA5, thereby supporting our initial hypothesis. The above experimental data preliminarily proved a rarely reported mechanism of adipocytes, anoikis, and ITGA5 in HNSCC progression.

However, our research still has several limitations that should be acknowledged. First, the study primarily relied on in vitro cell line models (FaDu and SAS), which may not fully recapitulate the complexity of the tumor microenvironment in patients. The use of additional cell lines or primary patient-derived cells could strengthen the generalizability of the findings. Second, although we demonstrated that adipocytes upregulate ITGA5 expression and promote anoikis resistance, the precise adipocytes-secreted molecular mediators that drive ITGA5 overexpression remain unknown. Potential candidates such as adipokines, exosomes, or metabolic factors require further investigation. Third, the clinical relevance of ITGA5 overexpression in HNSCC patients with adipose-rich environments has not been validated using patient’s samples or bioinformatic datasets.

To address these limitations, future studies should focus on the following directions: (1) Identifying the key adipocyte-derived factors that regulate ITGA5 expression, possibly; (2) Validating the association between adipocyte, ITGA5 expression, and anoikis resistance in clinical HNSCC specimens; (3) Utilizing patient-derived xenograft (PDX) models or genetically engineered mouse models to confirm the role of adipocyte-ITGA5 signaling in anoikis resistance and metastasis *in vivo*; (4) Exploring the potential of targeting ITGA5 or its downstream effectors as a therapeutic strategy to sensitize HNSCC cells to anoikis.

## Conclusion

In summary, our study demonstrates that adipocytes enhance anoikis resistance in HNSCC through upregulation of ITGA5. These findings not only provide mechanistic insight into the role of adipocytes in promoting HNSCC progression but also suggest that targeting the adipocyte-ITGA5 axis may represent a promising therapeutic approach for combating anoikis resistance in HNSCC.

## Data Availability

No datasets were generated or analysed during the current study.
